# Dengue Research Funded by the European Commission-Scientific Strategies of Three European Dengue Research Consortia

**DOI:** 10.1371/journal.pntd.0002320

**Published:** 2013-12-12

**Authors:** Thomas Jaenisch, IDAMS, Anavaj Sakuntabhai, DENFREE, Annelies Wilder-Smith, DengueTools

**Affiliations:** 1 Section Clinical Tropical Medicine, Department of Infectious Diseases, Heidelberg University Hospital, Heidelberg, Germany; 2 Unité de Génétique Fonctionnelle des Maladies Infectieuses, Institut Pasteur, Paris, France; 3 Department of Public Health and Clinical Medicine, Epidemiology and Global Health, Umeå University, Umeå, Sweden; Pediatric Dengue Vaccine Initiative, United States of America

Dengue is a major international public health concern and one of the most important arthropod-borne diseases [1]. Approximately 2.5 billion people—40% of the world's population, in over 100 countries—are at risk of dengue virus (DENV) infection [2]. In recent years the average annual incidence of dengue-related serious disease in many tropical counties has been rising dramatically, with the infection becoming endemic in areas where its occurrence was once sporadic [3].

The exponential increase over the last decade has been connected to societal changes, such as population growth and increasing urbanization [4]. In addition, it has been suggested that rising temperatures and global climate change may lead to the expansion of the range of major mosquito vectors into new areas, extension of the transmission season in current endemic areas, and increase in the mosquito species vectorial capacity [5]–[7]. Human migration (likely including infected hosts) and international travel are constantly introducing new vectors and pathogens into novel geographic areas [8]. For example, chikungunya virus was introduced into northeastern Italy in 2007, causing an outbreak with local transmission due to the presence of *Aedes albopictus*, a vector also capable of transmitting dengue virus [9]. In 2010, three authochthonous cases of dengue were reported in Europe, thereby highlighting the potential for global spread of this disease [10], [11]. The island of Madeira, where the mosquito vector *Aedes aegypti* is present, experienced a major dengue outbreak in the fall of 2012 [12], highlighting that the introduction of dengue to non-endemic areas is a real threat.

Dengue has been neglected for many years. Major research gaps for dengue exist in the areas of epidemiology under changing climate conditions, clinical management, pathogenesis, vector control, surveillance and response, vaccines, drugs, and health policy research [13].

The European Commission (EC) launched a call under the Seventh Framework Programme with the title of “Comprehensive control of Dengue fever under changing climatic conditions” (http://ec.europa.eu/research/participants/portal/page/cooperation?callIdentifier=FP7-HEALTH-2011-single-stage). The focus of this call is summarized in Box 1. Within this framework, in 2011, the EC awarded a total of approximately €18 million to three consortia. The hosting institutions are Heidelberg University Hospital (Germany), the Institute Pasteur (Paris, France), and Umeå University (Sweden). Each consortium has partners from countries with endemic and epidemic dengue. In total, the consortia comprise 38 partners from 21 countries, of which 11 are from Asia and Latin America, the current hotspots of dengue endemicity, and one from Africa (Figure 1).

**Figure 1:**
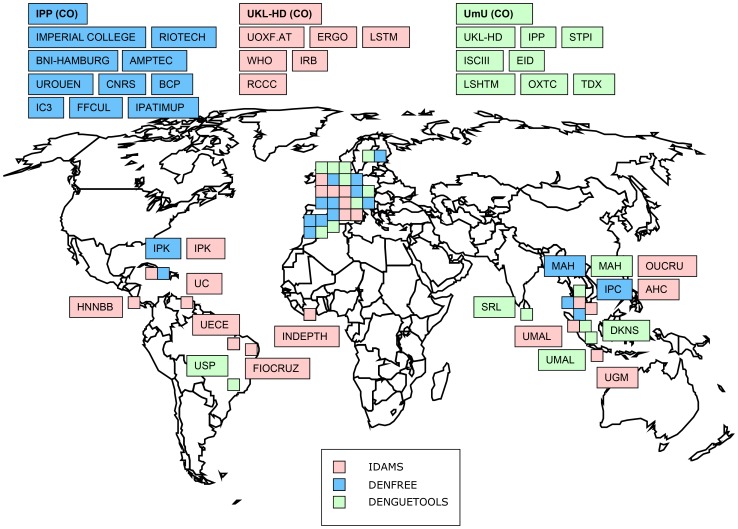
The world map of the three EU-funded dengue consortia. **IDAMS**: UKL-HD: Heidelberg University Hospital, Germany (Coordinator); UOXF.AT: Oxford University, UK; OUCRU: Oxford University Clinical Research Unit, Vietnam; LSTM: Liverpool School of Tropical Medicine, UK; IRB: Fondazione per l'Istituto di Ricerca in Biomedicina, Switzerland; WHO: World Health Organization - Special Programme for Training and Research in Tropical Diseases, Switzerland/Int. Partner; UMAL: University of Malaya Medical Center, Malaysia; UGM: University Gadjah Madah, Indonesia; AHC: Friends without a Border - Angkor Hospital for Children, Cambodia; IPK: Institute Of Tropical Medicine “Pedro Kouri”, Cuba; HNNBB: Ministry of Health - Hospital National de Ninos Benjamin Bloom, El Salvador; UECE: Fundacao Universidade Estadual do Ceara, Brazil; ERGO: Environmental Research Group Oxford Limited, UK; INDEPTH: INDEPTH-Network, Ghana; RCCC: Red Cross/Red Crescent Climate Centre, Netherlands; UC: University of Carabobo, Venezuela; FIOCRUZ: Fundacao Oswaldo Cruz, Brazil. **DENFREE**: IPP: Institut Pasteur, France (Coordinator); IMPERIAL COLLEGE: Imperial College, UK; BNI-HAMBURG: Bernard Nocht Institute, Germany; MAH: Mahidol University, Thailand; IPC: Institut Pasteur, Cambodia; IC3: Fundacio Institut Catala De Ciencies Del Clima, Spain; UROUEN: University Of Rouen, France; CNRS: Centre Nationale de la Récherche Scientifique, France; FFCUL: Cmaf, Fundacao Da Faculdade De Ciencias Da Universidade De Lisboa, Portugal; IPATIMUP: Instituto De Patologia E Imunologia Molecular Da Universidade Do Porto, Portugal; BCP: Biocomputing Platforms Ltd. Oy, Finland; AMPTEC: Amptec Ltd., Germany; RIOTECH: Riotech Pharmaceticals Ltd., UK; IPK: Institute Of Tropical Medicine “Pedro Kouri”, Cuba. **DENGUETOOLS**: UmU: Umea University, Sweden (Coordinator); SRL: Epidemiological Unit, Ministry of Health, Sri Lanka; TDX: TwistDx Ltd., Cambridge, UK; UMAL: University of Malaya, Malaysia; OXTC: Oxited Ltd., Oxford, UK; MAH: Mahidol University, Thailand; LSTMH: London School of Hygiene and Tropical Medicine, UK; STPH: Swiss Tropical and Public Health Institute, Switzerland; IPP: Institut Pasteur, France; UKL-HD: Heidelberg University Hospital, Germany; DKNS: Duke-NUS Graduate Medical School Singapore; USP: University of São Paulo, Brazil; ISCIII : Instituto de Salud Carlos III, Spain; EID: Entente Inter-Départementale pour la Démoustication du littoral méditerranéen, France. The two partners University of Carabobo (UC, Venezuela) and Fundacao Oswaldo Cruz (FIOCRUZ, Brazil) have been recently accepted to the IDAMS consortium. The amendment request submitted for approval to the European Commission is on-going.

**Box 1. European Commission Seventh Framework Programme FP7 Cooperation - Health**

**HEALTH.2011.2.3.3-2: Comprehensive control of Dengue fever under changing climatic conditions. FP7-HEALTH-2011-single-stage**

Research should develop innovative tools for one or more of the following aspects: better diagnosis, surveillance, development of treatment, prevention and vaccination strategies, prevention, and/or prediction and prevention of the spread of dengue fever to previously uninfected regions (including Europe), in the context of climate change. Research may also include studies on the underlying pathogenesis with respect to viral and host factors that can predict disease severity and prepare for further development of new vaccines, antiviral compounds, and more targeted treatment schemes.

**Funding Scheme** Specific International Cooperation Action (SICA) Collaborative Project (small- or medium-scale focused research project) target regions: Latin America and/or Asia. SICA aims to bring about the balanced participation of third countries in collaboration with European partners.

**Expected Impact** Better tools, and the use thereof, for improved comprehensive control of dengue fever at a global level. Participation from both SICA target regions and Small and Medium Enterprises SMEs in the projects should help ensure innovation and exploitation of the results in this area/topic. The degree of such participation will be considered during the evaluation.

Source: http://ec.europa.eu/research/health/infectious-diseases/emerging-epidemics/call-for-proposals_en.html

The funding of such a large and complex research programme focusing on a single disease highlights the emphasis that the European Commission has put on dengue and its potential threat to Europe.

In this paper, we present these three consortia and outline their scientific strategies and potential role within the international dengue research community.

## I. International Research Consortium on Dengue Risk Assessment, Management, and Surveillance (IDAMS)

### Rationale and Hypotheses

Differentiating dengue from other common febrile illnesses before complications develop is difficult; simple and inexpensive strategies are urgently needed to support early and accurate diagnosis, as well as to identify patients at high risk of developing complications. Similarly, characterisation of the profiles of important viral and serological biomarkers is likely to provide valuable information that could contribute to diagnostic and prognostic algorithms.

However, if the fight to gain control of the current global pandemic is to be successful it is equally important to consider interventions and strategies at the population level. Early detection of outbreaks, with improved surveillance systems and a prompt response to imminent outbreaks, could prove highly effective in reducing the numbers of dengue cases globally. In combination with identification of areas likely to be at risk of dengue outbreaks, as defined by risk mapping, such strategies could bring great health benefits.

### Description of Work Packages

The overall objectives of IDAMS are organized into six work packages (WP), grouped in two areas:

1. Improving clinical management and diagnosis of dengue (WP 1 & 2)
2. Assessing the risk of dengue spread (WP 3 & 4)

**Figure 2:**
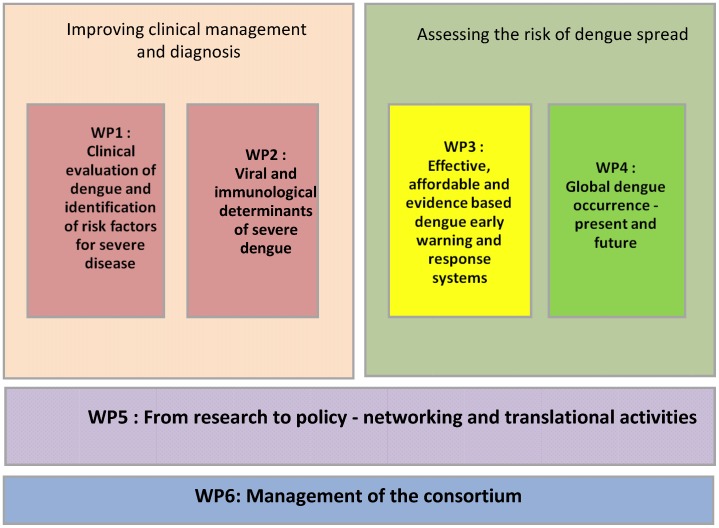
Structure of the work packages (WP) of the IDAMS consortium. The core of the IDAMS project focuses on parallel strategies aimed at: (1) Improving diagnosis and clinical management of dengue through two linked work packages designed a) to identify readily available clinical and laboratory parameters and/or viral and immunological markers that differentiate between dengue and other common febrile illness within three days of fever onset, and b) to identify any of the available markers that are predictive of the likelihood of evolving to a more severe disease course. (2) Assessing the risk of dengue spread though linked work packages focused on a) mapping and modeling techniques to define the current extent of dengue disease globally and to evaluate possible scenarios of spread or risk to previously uninfected regions in the future, and b) developing effective and affordable early warning and outbreak response systems. These four work packages are supported by a fifth work package dedicated to networking and translational activities to ensure that outputs from the various research activities are used to maximal advantage. A sixth work package will focus on administrative issues.

WP 5 & 6 are translational or administrative in character (Figure 2). In WP 1, a large prospective multicentre study has been designed aiming to improve the ability to distinguish dengue from other febrile illnesses in the early phase of disease, and to develop better prognostic markers or warning signs that predict the need for closer monitoring/hospitalisation, or are associated with development of severe disease. In addition, the clinical data will be used to assess the performance of the 2009 WHO classification in practice, and to inform the development of a more effective approach to dengue within the Integrated Management of Childhood Illness (IMCI) guidelines [14]. The prospective multicentre study is carried out in Vietnam, Cambodia, Malaysia, Indonesia, Brazil, Venezuela, and El Salvador.

The virological and immunological research in WP 2 builds on the infrastructure of the prospective clinical study with the intention of identifying virological, immunological, and host genetic variables associated with severe outcomes, and assessing their practical utility as prognostic markers. A novel approach to dengue vaccine development will also be explored via the generation of a T cell vaccine.

WP 3 will develop new country-specific models for early detection of dengue outbreaks together with better response mechanisms to minimise the consequences of such outbreaks; the best existing strategies will be employed alongside proven novel approaches and tools to effect these ends.

The mapping in WP 4 has two main aims: first, to develop a contemporary dengue occurrence map at the global scale [15]; and second, to investigate the potential impact of environmental change on this distribution and provide occurrence maps for 2020, 2050, and 2080.

WP 5 has an overarching role in IDAMS, providing a platform for networking and translational activities within the entire research programme by coordinating the input from network partners operating on a regional to global scale in the control of dengue (e.g., the Special Programme for Research and Training in Tropical Diseases of the World Health Organization [WHO-TDR]; the Red Cross/Red Crescent Climate Centre [RCCC]; the International Network for the Demographic Evaluation of Populations and Their Health [INDEPTH]; and the European Center for Disease Control [ECDC]). Part of this work will involve developing a research framework for dengue in Africa.

## II. Dengue Research Framework for Resisting Epidemics in Europe (DENFREE)

### Rationale and Hypotheses

Epidemiological studies have suggested that most dengue viral infections are subclinical or pauci-symptomatic—at least for primary dengue viral infections [16]–[18]. This means that in a completely naïve population, the first cases in hospitals will be the tip of the iceberg. Thus hospital-based surveillance is inadequate—too little and too late. Inherent in the DENFREE programme is the hypothesis that improved surveillance and diagnosis of the pauci-symptomatic dengue viraemic individuals will contribute to effective intervention. Active surveillance programmes to detect symptomatic infections include school-based absenteeism and community (door-to-door) approaches [19]. Neither of these approaches will be realistic or necessarily useful under an invasion scenario in Europe where the force of infection will be initially small. Whilst a programme of active surveillance would be ideal to detect completely subclinical infections, based on, for example, cluster analyses around index cases, this is not a realistic option and assumes dengue transmission is largely household based. Although there is good evidence that household transmission can occur, this is not always the case, as shown in Brazil [20] and suggested for the changing epidemiology of dengue in Singapore [21]. In Brazil, dengue cases clustered around places of high human movement and contact (e.g., markets). In Singapore, the effective intra-domiciliary vector control programmes reduced the importance of household transmission, but have remained ineffective at controlling overall dengue incidence, likely because of transmission hotspots in public places, including schools. Given the high level of access to health care in Europe, individuals with only very mild fever or other symptoms (pauci-symptomatic) are likely to present to their assigned General Practitioner (GP). Equipping a network of GPs with a simple diagnostic kit that can detect the virus even in pauci-symptomatic infections would provide a passive surveillance programme that can extend the detection of dengue infections beyond the more serious cases presenting at hospitals.

### Description of Work Packages

**Figure 3:**
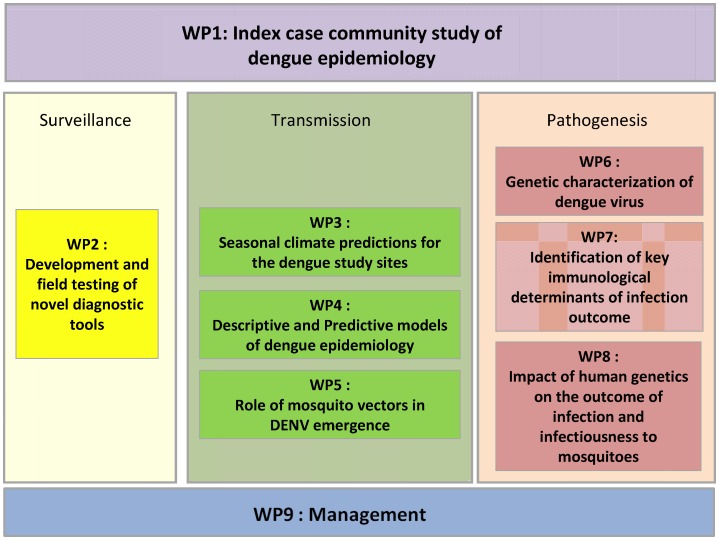
Structure of the work packages (WP) of the DENFREE consortium. For the DENFREE project work package (WP) 1 (Index case community study of the epidemiology of dengue) is a central WP, which will provide data and biological samples for other WPs. This WP is a multicentric, prospective study in Cambodia and Thailand, which will characterize local DENV transmission patterns, identify subclinical infections for mosquito transmission studies (WP5 entomology), establish empirical mosquito, human density, and geo-spatial data for use in fine-scale and agent-based simulation models (WP3 climate prediction and WP4 epidemiological models), establish a biobank of biological samples from patients, household members, and mosquito vectors for further study in other WPs (WP2 diagnostics, WP6 virology, WP7 immunology, and WP8 human genetics), and test novel diagnostic and prognostic tools developed by WP2. Contributions from each WP will bring complementary help to the consortium to achieve the main aims. WP2 will develop new point-of-care diagnostic tools that can be used in the community to screen subclinical individuals in epidemic regions and to test for DENV in mosquito samples, thereby validating a new mosquito trap tool developed by WP5. WP3 and WP4, by using better surveillance data, will help determine the underlying factors, extent, and course of a DENV epidemic. Altogether, we will provide a new strategy for dengue surveillance for better control of DENV transmission.

The work programme is broken down into nine work packages with one work package (WP 9) dedicated to consortium management, assessment of progress, and dissemination of the results (Figure 3).

WP 1 (Index case community study of the epidemiology of dengue) is a central WP, which will provide data and biological samples to other WPs. They will characterize local dengue virus (DENV) transmission patterns through cluster analyses [22], identify subclinical infections for mosquito transmission studies (WP 5 entomology), by direct and indirect feeding to laboratory-reared mosquitoes, establish empirical mosquito, human density, and geo-spatial data for use in fine-scale and agent-based simulation models (WP 3 climate prediction and WP 4 epidemiological models), establish a biobank of biological samples from patients, household members and mosquito vectors for further study in other WPs (WP 2 diagnostics, WP 6 virology, WP 7 immunology, and WP 8 human genetics), and test novel diagnostic and prognostic tools developed by WP 2 in retrospective samples and prospective recruited cohorts in Cambodia and Thailand.

## III. Innovative Tools and Strategies for the Surveillance and Control of Dengue (DengueTools)

### Rationale and Hypotheses

We lack good understanding of individual or combined roles of viral, entomological, ecological, environmental, and climate factors that influence dengue transmission dynamics and their respective outbreak predictive capability and the most cost-effective approach for surveillance and early warning systems. For surveillance to effectively provide early warning for epidemic transmission, it must be active, laboratory-based, and comprehensive in its coverage of the spectrum of clinical illness and the factors that influence transmission dynamics. Several potential predictive indicators for outbreaks have been described but require further study [23], [24]. Early diagnostic assays at point-of-care that are affordable and can be used in the field are also missing. Vector surveillance in particular has many shortcomings, specifically the lack of sensitive, reliable, and simple field methods for vector surveillance.

Furthermore, in many endemic countries, children are the most affected group, in terms of both incidence and severity of dengue. Effective control strategies to protect children are lacking—in particular, simple, cost-effective, and scalable strategies. Children spend a substantial amount of their time at schools, and *Aedes* mosquitoes bite mainly during the day. Although transmission is reported to occur at the household level, there is also increasing evidence that schools may contribute to transmission [25].

We hypothesize that insecticide-treated school uniforms may be a target for a school-based intervention to reduce the incidence of dengue in school children [26], and propose to test this hypothesis under laboratory and field conditions.

Lastly, the risk of introduction of dengue to non-infected areas, including Europe, needs to be explored in more detail in order to enhance Europe's preparedness for the potential emergence of dengue. We currently have insufficient data on the magnitude and trends of importation and virus evolution over time and by geographic origin. We also only have a poor understanding of vector density, preferred breeding sites, and vectorial capacity of *Aedes* in temperate climates that are needed for predictive models under changing climate conditions.

### Description of Work Packages

The DengueTools consortium research strategy has been described in more detail elsewhere [27]. In summary DengueTools has developed three research areas to address the above outlined gaps related to (1) surveillance, (2) prevention, and (3) risk of introduction to uninfected areas.

**Figure 4:**
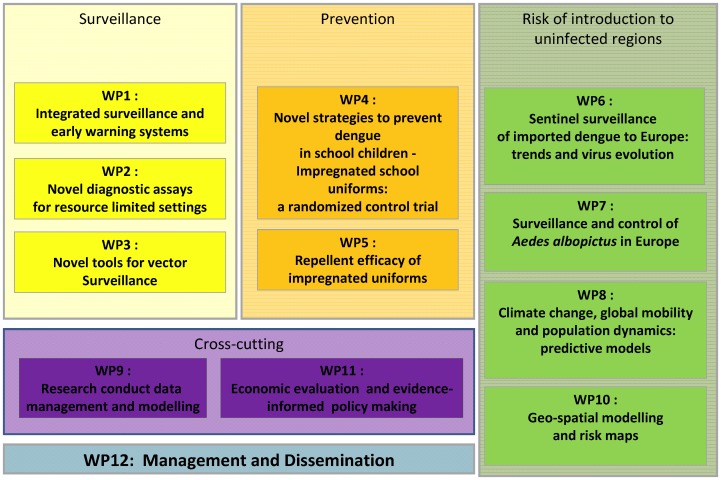
Structure of the work packages (WP) of the DengueTools consortium. The DengueTools project is comprised of 12 work packages around the following three research areas: **Research area 1**: Develop a comprehensive early warning and surveillance system that has predictive capability for epidemic dengue and benefits from novel tools for laboratory diagnosis and vector monitoring. **Research area 2**: Develop novel strategies to prevent dengue in children. **Research area 3**: Understand and predict the risk of global spread of dengue, in particular the risk of introduction and establishment in Europe, within the context of parameters of vector competence, global mobility, and climate change.

DengueTools consists of 12 work packages around these three research areas (Figure 4).

Research area one with three work packages focuses on surveillance with the objective to develop a comprehensive early warning and surveillance system that has predictive capability for epidemic dengue and benefits from novel tools for laboratory diagnosis and vector monitoring. WP 1 plans to set up a comprehensive, early warning, laboratory-based sentinel disease surveillance system in Sri Lanka. This prospective study will use an integrated surveillance system that incorporates a set of indicators that include clinical, epidemiologic, virologic, entomologic, meteorologic/climate, environmental, and socio-economic data. The intent is to identify factors, or a combination of factors, that most sensitively predict epidemic dengue. WP 2, based in Kuala Lumpur, Malaysia, partnering with TwistDx in Cambridge, UK, has set out to develop two novel approaches for a point-of-care early diagnostic assay that can potentially be used in field conditions and surveillance systems. Oxitec in Oxford, UK, is responsible for WP 3, which will evaluate novel approaches to vector surveillance.

Research area 2 with two work packages, focuses on the prevention of dengue in school children using insect repellent–impregnated school uniforms. Partners in WP 4 will conduct a school-based randomized controlled trial in ten schools in Chochaengsao Province, Thailand. The trial protocol is publicly available [28]. To complement this trial, we will also conduct laboratory-based studies on impregnated school uniforms at the London School of Hygiene & Tropical Medicine (WP 5).

Research area 3 with another three work packages focuses on the risk of introduction of dengue into currently non-endemic areas. WP 6 will collect clinical and virological data in travelers returning to Europe from dengue-endemic countries. WP 7 has set out to explore the role of *Aedes albopictus* in southern France, but will also investigate control strategies of *Aedes albopictus* in Europe. WP 8 will work on predictive risk modeling and maps under different future climate scenarios in Europe.

Cross-cutting work packages were added with skills and expertise in research conduct and data management (WP 9), geo-spatial modeling and risk maps (WP 10), and economic evaluation and evidence-informed policy making (WP 11), mainly contributing to WP 1 and WP 4, but also to other work packages if needed.

Lastly, WP 12 is responsible for management and dissemination of the scientific results.

## Summary and Outlook

To respond to the call under the Seventh Framework Programme with the title of “Comprehensive control of Dengue fever under changing climatic conditions,” each of the three consortia developed programmes that address many of the major research gaps as identified by the European Commission (Box 1). Box 2 lists the deliverables that each of the consortia has set out to achieve.

**Box 2. Deliverables of the Three European Commission-Funded Dengue Research Consortia**

**I. International Research Consortium on Dengue Risk Assessment, Management, and Surveillance (IDAMS):**

- Development of study Case Report Form (CRF) and initiation of study at first site
- Development of centralised computer database for local data entry at each site
- Recruitment of 50% of sample size with data entry completed
- Evaluation report on warning signs and on the practical application of the 2009 WHO dengue case classification scheme, compared with the DHF/DSS classification scheme
- Molecular diagnosis of dengue patients underway
- Serological assays optimised and characterised
- Characterization of the immunogenicity of novel vaccine constructs
- Identification of reliable, sensitive, practical epidemiological indicators
- Recommendations for outbreak response tools for effective vector control tools
- Recommendations for outbreak response plans for health care service reorganisation
- Integrated Surveillance Model
- Contemporary/baseline map of dengue occurrence
- Future map of dengue occurrence (2020, 2050, and 2080)
- Consensus documents on (1) research process and (2) policy recommendations
- Policy briefs on the organisation of dengue surveillance
- Documented quality assurance in all WPs

**II. Dengue Research Framework for Resisting Epidemics in Europe (DENFREE)**

- Evaluate the impact of climate on the incidence of disease in Southeast Asia
- Develop dynamical models that incorporate both short-term protective cross-immunity and longer term antibody-dependent enhancement to explain the incidence of dengue observed in Thailand over the last 30 years
- Integrate a dynamic disease model within a seamless ensemble prediction system and assess the extent to which climate-variable forecasting can usefully explain dengue incidence
- Develop real-time agent based models pinpointing key factors determining dengue diffusion at the very local scale
- Estimate proportion of inapparent dengue viral infections that result in mosquito transmission
- Evaluate vector competence in European mosquito species
- Evaluate an alternative tool to reduce dengue risk at pauci-symptomatic dengue viremic carriers
- Identify viral genetic markers correlating with subclinical/symptomatic infection
- Identify viral genetic markers associated with enhanced transmission by the European mosquito *Aedes albopictus*
- Perform a comparative analysis of the immunological response (B cell, T cell, and cytokine) to infections that have lead to subclinical and symptomatic infections
- Evaluate role of human immune-related genes in the outcome of dengue viral infection
- Evaluate new anti-dengue viral compounds: pharmacokinetics, toxicities, and activity against dengue replication and immunological consequences *in vivo* in mouse models
- Develop an ultra-sensitive tool to detect virus
- Develop a simple and rapid test for detection of DENV antibodies on a dipstick platform
- Develop a test for detection of DENV specific immune responses on an antigen array platform

**III. Innovative Tools and Strategies for the Surveillance and Control of Dengue (DengueTools)**

- Develop and validate novel diagnostic assays for point-of-care use
- Field-test novel diagnostic assays
- Develop novel field devices and attractants for vector monitoring
- Develop novel assays for virus detection and characterisation in *Aedes* mosquitoes
- Develop geospatial modeling and risk maps
- Develop a comprehensive, early warning, laboratory-based sentinel disease surveillance system
- Study viral genomic sequence data and the potential role in causing outbreaks of more severe disease
- Evaluate an integrated surveillance system and identification of the most useful and cost-effective outbreak predictive factors, or the combination thereof
- Develop early warning predictive models, in particular signature forecasting and a flagging system
- Engage policymakers on the sustainability of an integrated surveillance system
- Determine the protection efficacy of school uniforms impregnated with repellent under different laboratory scenarios
- Determine the efficacy of impregnated school uniforms on the reduction of dengue incidence in school-aged children
- Develop a cost-effectiveness framework and model for the school-based preventive intervention
- Produce policy briefs to ensure the scalability of such school intervention programmes
- Describe the trends of dengue importation into Europe
- Produce phylogenetic trees and Bayesian modeling on dengue viruses imported to Europe
- Identify typical breeding sites for *Ae. albopictus* in southern France
- Evaluate the impact of ultra-low volume (ULV) aerosols on the abundance of *Ae. albopictus* in southern France
- Estimate values for the parameters of vectorial capacity
- Develop predictive models that integrate vectorial capacity to assess the risk of establishment of dengue following introduction of the virus

The three consortia each have a different focus—but in some aspects also common themes. Table 1 summarizes the commonalities and differences.

**Table 1:** Overlap and complementarity of research areas between the three EU-funded dengue consortia.

Research areas	IDAMS	DengueTools	DENFREE
Assessing the risk of dengue spread	WP3, WP4		
Geo-spatial modeling and risk maps	WP4	WP 10	
Innovative tools for prediction and prevention of dengue spread			WP3, WP4, WP5
Risk of introduction to uninfected regions		WP6, WP7, WP8	
Surveillance	WP3	WP1, WP2, WP3, WP6	WP2, WP5
Prevention	WP3	WP4, WP5	
Improving clinical management and diagnosis	WP1, WP2	WP1	WP1
Diagnostic tools	WP1, WP2	WP2	WP2
Virology	WP2		WP6
Immunology	WP2		WP7
Human genetics/mouse model	WP2		WP8
Economic evaluation	WP4	WP11	
Evidence-informed policy making	WP3	WP11	
Networking and translational activities	WP5	WP12	
Research conduct, data management and modeling	WP1	WP9	
Management	WP6	WP12	WP9

All three consortia propose to use an evidence-based multidisciplinary approach to identify the key combinations of factors predictive of dengue outbreaks in endemic settings. The common objective is to identify innovative tools, develop new strategies for dengue surveillance for early detection and effective response to the threat of outbreaks, and develop dengue risk maps on a global scale.

Maps can be powerful tools for advocacy, but have also been proven useful to investigate the equity and adequacy of international financing—e.g., in the case of malaria control [29]. IDAMS will undertake a global mapping programme to address the true extent of the global distribution of dengue in order to provide a basis upon which future risk and burden of disease can be addressed [30]. This includes prediction and prevention of the spread of dengue fever to previously uninfected areas—an area also of importance for DengueTools. DengueTools places particular emphasis on dengue risk maps under different climate and future scenarios, influenced by global human mobility. DENFREE places more emphasis on the transmissibility of the virus according to the clinical state of the infected human (i.e., subclinical vs. symptomatic) and the vector–virus genetic constitution.

To address research questions around the evolving epidemiology of dengue, the approaches of the three consortia are quite different: While IDAMS uses comparative country studies based on existing data and consultation of expert committees, then performs prospective evaluation of the improved model in key locations, DENFREE implements a community-based approach, with emphasis on studying the spread of dengue at the local scale, examining the importance of micro-scale environmental parameters on dengue epidemiology and addressing the extent and epidemiological importance of pauci-symptomatic and/or subclinical infections. DengueTools implements a prospective study design to better determine the most appropriate and cost-effective dengue surveillance system using a comprehensive, active, laboratory-based approach.

Also in areas other than mapping, the consortia have outlined similar or complementary research objectives. Both IDAMS and DENFREE will investigate methods for risk factors and markers that may predict the development of more severe disease, which would be important information for better clinical case management at the individual patient level. For clinical, virological, and host factors predicting disease severity, IDAMS focuses on a large cohort study in outpatient facilities and hospitals around the world in order to identify factors associated with the development of severe disease. DENFREE plans to collect information of dengue in the community, ranging from subclinical infection to clinical dengue. DengueTools plans to look at clinical and virological parameters that may contribute to improved surveillance systems.

Both DENFREE and DengueTools propose to develop a point-of-care dengue diagnostic test and novel tools for vector monitoring. In addition, DengueTools will conduct a community-based intervention trial to investigate a novel approach to prevent dengue in school children by using insect repellent-impregnated school uniforms.

Despite the different research strategies of the three EU-funded consortia, some similar approaches allow for collaborative efforts. Supported by the EC, the three consortia met in April 2012 in order to discuss synergies and a common agenda. As a result of this meeting, the three EC-funded consortia will collaborate on a global risk map for dengue to enhance dengue control and facilitate the communication about the continuing threat of dengue spreading to previously uninfected areas.

In conclusion, dengue is currently high on the political agenda of many endemic areas, particularly of those countries that have experienced a recent dramatic rise in incidence. Funding of three dengue-focused consortia by the European Commission also highlights Europe's interest in this disease. The three publicly funded dengue research consortia present a unique opportunity to address the threat of dengue in endemic countries, but also to Europe. It will be important for these consortia to reach out to the global players in the international scientific dengue community. It is equally important for existing international and national dengue research groups to engage with newer dengue research groups such as these three EU-funded consortia. One example for a platform to enhance international scientific networking and exchange is the Global Health Network. The Global Health Network is a collection of websites that aim to support research by sharing knowledge and methods (http://tghn.org/). The ultimate aim is to build collaborations, share resources, and exchange information.
