# Correction: Dengue Research Funded by the European Commission-Scientific Strategies of Three European Dengue Research Consortia

**DOI:** 10.1371/journal.pntd.0002883

**Published:** 2014-04-18

**Authors:** The PLOS Neglected Tropical Diseases Staff

In the Acknowledgments section, the affiliation of Xavier Rodó is listed incorrectly. The correct affiliation is: ICREA and Institut Català de Ciències del Clima.
